# Failure of Fluid Absorption in the Endolymphatic Sac Initiates Cochlear Enlargement that Leads to Deafness in Mice Lacking *Pendrin* Expression

**DOI:** 10.1371/journal.pone.0014041

**Published:** 2010-11-17

**Authors:** Hyoung-Mi Kim, Philine Wangemann

**Affiliations:** Anatomy and Physiology Department, Kansas State University, Manhattan, Kansas, United States of America; Hospital of the University of Pennsylvania, United States of America

## Abstract

Mutations of *SLC26A4* are among the most prevalent causes of hereditary deafness. Deafness in the corresponding mouse model, *Slc26a4^−/−^*, results from an abnormally enlarged cochlear lumen. The goal of this study was to determine whether the cochlear enlargement originates with defective cochlear fluid transport or with a malfunction of fluid transport in the connected compartments, which are the vestibular labyrinth and the endolymphatic sac. Embryonic inner ears from *Slc26a4^+/−^* and *Slc26a4^−/−^* mice were examined by confocal microscopy *ex vivo* or after 2 days of organ culture. Culture allowed observations of intact, ligated or partially resected inner ears. Cochlear lumen formation was found to begin at the base of the cochlea between embryonic day (E) 13.5 and 14.5. Enlargement was immediately evident in *Slc26a4^−/−^* compared to *Slc26a4^+/−^* mice. In *Slc26a4^+/−^* and *Slc26a4^−/−^* mice, separation of the cochlea from the vestibular labyrinth by ligation at E14.5 resulted in a reduced cochlear lumen. Resection of the endolymphatic sacs at E14.5 led to an enlarged cochlear lumen in *Slc26a4^+/−^* mice but caused no further enlargement of the already enlarged cochlear lumen in *Slc26a4^−/−^* mice. Ligation or resection performed later, at E17.5, did not alter the cochlea lumen. In conclusion, the data suggest that cochlear lumen formation is initiated by fluid secretion in the vestibular labyrinth and temporarily controlled by fluid absorption in the endolymphatic sac. Failure of fluid absorption in the endolymphatic sac due to lack of *Slc26a4* expression appears to initiate cochlear enlargement in mice, and possibly humans, lacking functional *Slc26a4* expression.

## Introduction

Mutations of *SLC26A4* are worldwide among the most prevalent causes of deafness [1], [2], [3], [4]. Particularly in Chinese populations, mutations of *SLC26A4* are found in as many as 13.7% of deaf subjects [5]. Phenotypes associated with mutations of *SLC26A4* include deafness at birth as well as fluctuating hearing loss that progresses toward deafness during childhood [6], [7], [8]. The compatibility of *SLC26A4* mutations with hearing, although limited to early childhood, provides the imperative to investigate the etiology of *SLC26A4* related deafness with the ultimate goal to develop strategies to preserve hearing in afflicted individuals. Toward this goal, the first mouse model, *Slc26a4^−/−^* (formerly named *Pds^−/−^*), had been developed [9]. Studies using this mouse model have mainly focused on the postnatal development since normal mice acquire hearing during the second postnatal week and since most human patients lose hearing during early childhood. Three synergistic pathophysiological pathways have so far been delineated.

**Figure 1:**
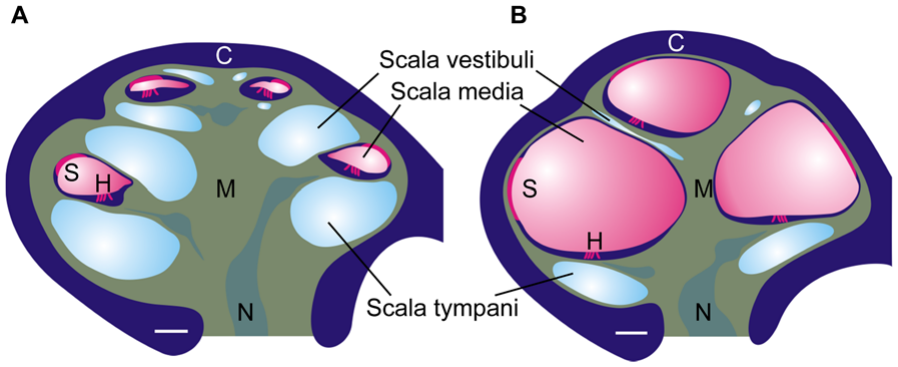
Schematic diagram of the cochlea. A) Diagram based on a cochlea obtained from an E18.5 *Slc26a4^+/−^* mouse. B) Diagram based on a cochlea obtained from an E18.5 *Slc26a4^−/−^* mouse, see Fig. 3. Abbreviations: C, otic capsule; S, stria vascularis; H, sensory hair cells; M, modiolus; N, cochlear nerve. Note, that spaces occupied by mesenchymal cell (green) are compressed in *Slc26a4^−/−^* mice. Particular, fibrocytes in the modiolus (M) and between the otic capsule (C) and stria vascularis (S) are displaced.

1) Lack of pendrin, the protein coded for by the gene *Slc26a4*, leads to a ∼10-fold enlargement of scala media [9], [10]. Scala media, is an endolymph-filled luminal space that is lined by the cochlear epithelium, which includes both the sensory hair cells as well as stria vascularis (Fig. 1A). Stria vascularis secretes K^+^ into endolymph and generates the endocochlear potential that provides the majority of the driving force for the sensory transduction, which occurs in the hair cells. Enlargement of scala media leads to hampered cell-to-cell communication and disruption of development toward hearing [10].

2) The enlargement of scala media forces stria vascularis in the postnatal cochlea to elevate rates of K^+^ secretion in order to maintain a normal endolymphatic K^+^ concentration [11]. Oxidative stress ensues from increased transport and leads to a loss of the K^+^ channel *Kcnj10*, which generates the endocochlear potential [12], [13], [14]. Loss of *Kcnj10* leads to a loss of the endocochlear potential and thereby of the driving force necessary for hearing.

3) The enlargement of scala media and the associated elevated rates of K^+^ secretion lead to an enhanced metabolic acid production by marginal cells of stria vascularis that is partially released into endolymph. Acid release into endolymph in conjunction with a loss of pendrin-mediated HCO_3_^−^ secretion lead to a luminal acidification by ∼0.3 pH-units [12]. Acid-mediated inhibition of Ca^2+^ absorption results in a ∼100-fold elevation in the endolymphatic Ca^2+^ concentration in *Slc26a4^−/−^* mice compared to *Slc26a4^+/−^* mice [12]. Excessive Ca^2+^ concentrations in endolymph impair sensory transduction by damaging outer hair cells through excessive Ca^2+^ influx via the apical transduction channel and subsequent cellular Ca^2+^ overload [9], [15].

From these studies in the mouse model, it is evident that the enlargement of scala media is a key event in the etiology of deafness. Most likely, the enlargement is due to a dysfunction of epithelial fluid transport, since *Slc26a4* codes for pendrin, which is an epithelial anion exchanger and since the enlargement occurs shortly after the onset of expression [9]. Conceptually, the enlargement could be the result of a net increase in fluid secretion or decrease in absorption. Pendrin itself does not mediate net solute transport, since it transports anions with a stoichiometry of 1∶1 [16]. Whether and how pendrin contributes to fluid secretion or absorption is unknown. This question is complicated by two issues:

First, virtually nothing is known about ion and fluid transport in the embryonic cochlea, which is when the enlargement develops. The adult ion composition, characterized by a high K^+^ concentration and low Na^+^ and Ca^2+^ concentrations, is established during postnatal development [12], [17], [18]. This means that investigations of ion transport in the postnatal cochlea cannot be extrapolated to the early embryonic stage when the cochlear lumen first develops.

Second, the epithelial enlargement in *Slc26a4^−/−^* mice is not limited to the cochlea. The vestibular labyrinth and the endolymphatic sac are prominently enlarged, too [9]. Interestingly, these compartments are connected to the cochlea and each contains pendrin-expressing epithelial cells. The role of pendrin in fluid transport may be different in the different compartments and it is conceivable that one compartment ‘pumps up’ or ‘drains’ the others. Thus, the goal of the present study was to determine the onset of cochlear enlargement and to investigate whether the enlargement of the cochlea in *Slc26a4^−/−^* mice is due to a local cochlear dysfunction or whether the cochlear enlargement is caused by dysfunction of the adjacent vestibular labyrinth or endolymphatic sac.

## Methods

### Animals

A colony of *Slc26a4^−/−^* and *Slc26a4^+/−^* mice was maintained at Kansas State University. Pairs of *Slc26a4^+/−^* dams and *Slc26a4^−/−^* sires were housed together. Litters sizes averaged 5.1 pups with *Slc26a4^+/−^* and *Slc26a4^−/−^* offspring in the Mendelian ratio of 50.1 to 49.9. The gestational period was 21 days. Genetic drift was limited by occasional back-crossing to the original strain, 129SvEvTac, that is maintained by Taconic, Germantown, NY. The colony was maintained free of known and suspected murine pathogens. Serologic tests (Radil, Columbia, MO), that were consistently and repeatedly negative, included the following antibodies: EDIM (Epizootic diarrhea of infant mice virus - a mouse rotavirus), TMEV (Theiler's murine encephalomyelitis virus - mouse poliovirus, strain GDVII), MHV (Mouse hepatitis virus - a mouse coronavirus), MVM (Minute virus of mice - a mouse parvovirus), MNV (Murine norovirus - a mouse calicivirus), M. pulmonis (Mycoplasma pulmonis – the agent of murine mycoplasmosis), MPV (a mouse parvovirus), Parvo NS-1 (a conserved recombinant parvoviral protein, rNS1) and Sendai (Sendai virus – a type 1 paramyxovirus).

*Slc26a4^+/−^* and *Slc26a4^−/−^* mice ranging from E11.5 to P7 were used for the present study. Time-pregnant dams were deeply anesthetized with 4% tri-bromo-ethanol and sacrificed by decapitation after harvesting embryos by sterile laparotomy. Gestational age was counted from the day, when a vaginal plug was detected. This day was set to embryonic (E) day 0.5 (E0.5). Gestational age, however, was verified, and in rare cases corrected, by evaluating gross morphological features including limbs, digits, and appearance of the pinna and auditory meatus [19], [20]. Neonatal mice (P0-P3) were anesthetized by a combination of i.p. injection of 0.013 ml/g body weight of 4% tri-bromo-ethanol and rapid cooling on an ice slush. Older mice (P5-P7) were anesthetized solely by i.p. injection of 0.013 ml/g body weight of 4% tri-bromo-ethanol. Embryos and postnatal mice were sacrificed by decapitation. All procedures involving animals were approved by the Institutional Animal Care and Use Committee of Kansas State University (IACUC#: 2613).

### Organ culture

Procedures for organ culture were adapted with modifications from methods developed by Dr. Thomas van de Water [21]. Embryos were harvested at E14.5 and E17.5. Otocysts were isolated in sterile NaCl solution maintained at 4°C. NaCl solution contained (in mM) 150 NaCl, 5 HEPES, 3.6 KCl, 1 CaCl_2_, 1 MgCl_2_ and 5 glucose, pH 7.4. The cranium was opened medially and otocysts were isolated after removal of the cerebrum.

Otocysts were left intact or altered either by ligation of the vestibular labyrinth or by resection of the endolymphatic sac. One otocyst from each embryo was left intact and the other was altered to facilitate paired comparisons. In one set of experiments, the cochlea was isolated from the vestibular labyrinth by ligation. Otocysts were isolated and tied at the base of the cochlea with a braided silk surgical suture (size 10-0, Deknatel, Queens Village, NY). After tying off the cochlea, the vestibular part of the otocyst, including the endolymphatic sac, was cut away from the isolated cochlear preparation. In another set of experiments, the endolymphatic sac was removed by resection. The endolymphatic sac was carefully peeled off the otocyst. Great care was taken in this set of experiments to not injure the endolymphatic sac, particularly the distal end, which is located in the posterior cranial fossa within the dura.

Otocysts, altered or intact, were maintained in organ culture for two days. Otocysts were placed into 12-well plates (Cat# 353503, Fisher, St. Louis, MO), submersed in DMEM/F-12 media (Cat# 12500-062, Invitrogen, Carlsbad, CA) supplemented with 10% fetal bovine serum (Cat# 30-2020, ATCC, Waldorf, MD) and 1% penicillin-streptomycin (Cat# 15140-122, Invitrogen) and incubated at 37°C in a humidified atmosphere enriched with 5% CO_2_ (HeraCell 240, Heraeus, Germany). Media was changed every 24 hrs.

### Confocal immunocytochemistry

Freshly isolated or cultured otocysts were fixed at 4°C for 2 hrs in a solution containing (in mM) 150 NaCl, 3.6 KCl, 5 HEPES, 1 CaCl_2_, 1 MgCl_2_ and 5 glucose, pH 7.4 and 4% paraformaldehyde. Fixed otocysts were processed through a sucrose gradient (10% and 20%, each 20 min, followed by 30% overnight, all at 4°C), infiltrated with polyethylene glycol (Cat# 72592-B, Electron Microscopy Sciences, Hatfield, PA) and cryo-sectioned (12 µm, CM3050S, Leica, Germany). Serial sections throughout the entire cochlea were obtained from embryos between ages E11.5 and E14.5 and mid-modiolar sections of the cochlea were obtained from embryos between ages E15.5 and E18.5 and from neonates aged P3.

Sections mounted on charged slides (Cat#22-230-900, Fisher) were blocked for 1 hr with 5% bovine serum albumin (BSA) in PBS-TX containing (in mM) 137 NaCl, 2.7 KCl, 10.1 Na_2_HPO_4_, 1.8 KH_2_PO_4_, pH 7.4, and 0.2% Triton-X-100. For morphological observations, sections were stained for actin filaments (Alexa488 conjugated phalloidin, Invitrogen) and for nuclei (DAPI, Invitrogen). Slides were incubated for 1 hr at room temperature with phalloidin and DAPI diluted with PBS-TX at 1∶40 and 1∶1,000, respectively. For immunocytochemistry, slides were incubated overnight at 4°C with primary antibody (rabbit anti-Na^+^/K^+^ ATPase 1 alpha subunit, Novus Biologicals, Littleton, CO) diluted 1∶200 with PBS-TX containing 1–3% BSA. Slides were washed three times in PBS-TX and incubated for 1 hr at room temperature with secondary antibody (Alexa594 conjugated goat-anti-rabbit (Invitrogen) diluted 1∶1,000 with PBS-TX containing 1–3% BSA. Stains for actin filaments (Alexa488 conjugated phalloidin (Invitrogen) and for nuclei (DAPI) were added to the secondary antibody at dilutions 1∶40 and 1∶1,000, respectively. After staining, slides were washed three times with PBS-TX, cover-slipped with FluorSave (Calbiochem, La Jolla, CA) and observed by confocal laser scanning microscopy (LSM 510 Meta, Carl Zeiss, Göttingen, Germany). The size of the cochlear lumen was evaluated by digital area measurements using image analysis software (Carl Zeiss).

### Statistics

Data are generally presented as average ± sem with n being the number of animals. Data acquired in paired experiments using littermates were evaluated by paired t-test. Significance was assumed when p<0.05.

## Results

### Cochlear lumen formation

**Figure 2:**
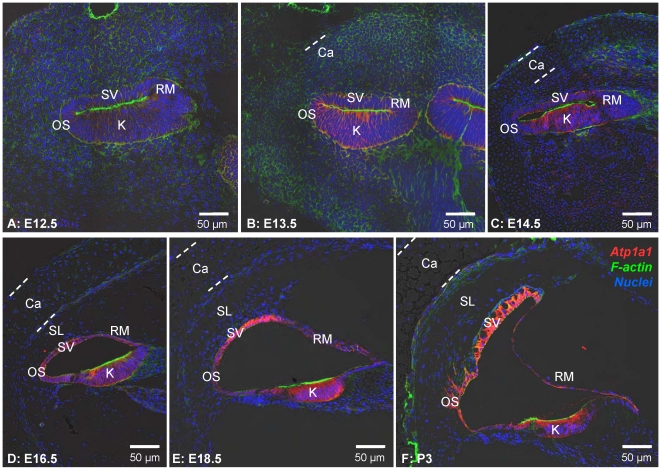
Cochlear development from E12.5 to P3 in *Slc26a4^+/−^* mice. Na^+^/K^+^ ATPase (red) was visualized by immunocytochemistry. F-actin (green) and nuclei (blue) were labeled. A-F: Six different stages of development ranging from E12.5 to P3 are shown. Abbreviations: SV, stria vascularis; OS, outer sulcus; K, Kölliker's organ; RM, Reissner's membrane; Ca, otic capsule; SL, spiral ligament. The thickness of the otic capsule is marked by dashed lines.

**Figure 3:**
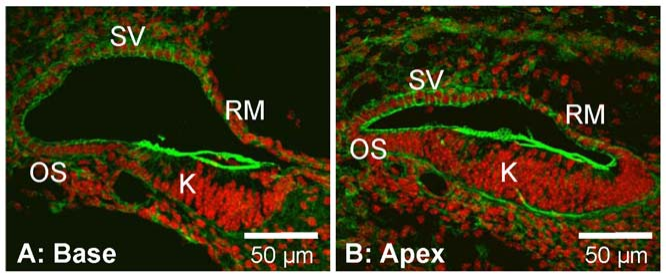
Cochlear lumen formation progressed from the base to the apex. A: Base of the cochlea. B: Apex of the same cochlea. F-actin (green) and nuclei (red) were labeled. Abbreviations: SV, stria vascularis; OS, outer sulcus; K, Kölliker's organ; RM, Reissner's membrane.

In a first set of experiments, we determined the onset of lumen formation in the cochlea. Inner ears ranging from embryonic (E) day E12.5 to postnatal (P) day P3 were sectioned and examined by confocal microscopy. At E12.5 and E13.5, the cochlear duct consisted of a closed epithelial tube (Fig. 2). Köllikers organ, the precursor of the organ of Corti, was evident on the basal side of the epithelial tube as a multilayered epithelium with an actin-rich region near the apical membrane. Köllikers organ was clearly distinct from the precursors of stria vascularis, outer sulcus and Reissner's membrane. At E13.5, the otic capsule was beginning to form. At E14.5, a clear distinction became evident between chondrocytes comprising the otic capsule and mesenchymal cells surrounding the cochlear epithelium. Further, the cochlear epithelium began to form a lumen. Around E16.5, the characteristic triangular cross section of the cochlea began to develop. At E18.5, stria vascularis began to differentiate from a monolayer to the multi-layered architecture including the characteristic vascularization. Lumen formation progressed from the base to the apex of the cochlea (Fig. 3).

### Cochlear enlargement occurs prior to cochlear *Slc26a4* expression

**Figure 4:**
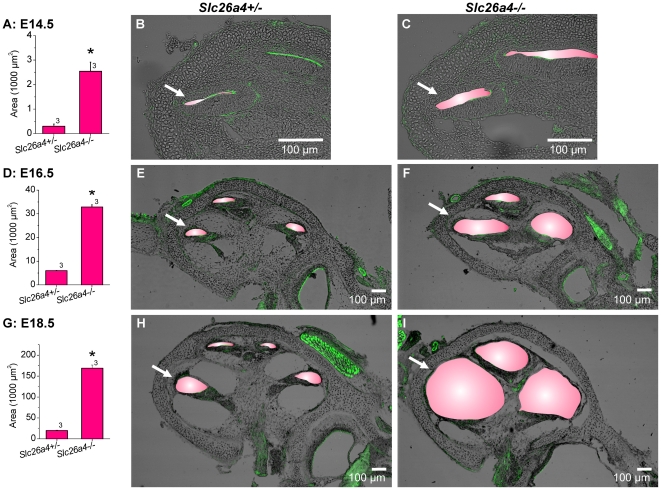
Cochlear lumen formation in *Slc26a4^+/−^* and *Slc26a4^−/−^* mice. A, D and G: Luminal area measurements of cross-section #1 in cochleae obtained from E14.5, E16.5 and E18.5 littermates, respectively. The number next to the bars represents the N number of animals. Significant differences are marked (*). B and C, cochlear cross-sections of E14.5 *Slc26a4^+/−^* and *Slc26a4^−/−^* littermates. E and F, cochlear cross-sections of E16.5 *Slc26a4^+/−^* and *Slc26a4^−/−^* littermates. H and I, cochlear cross-sections of E18.5 *Slc26a4^+/−^* and *Slc26a4^−/−^* littermates. Scala media was highlighted in pink. Images without the pink overlay are available in Figure S1 (Supporting information). Arrows point to cross-section #1, which was used for digital area measurements. F-actin (green) was labeled. Cochlear cross-sections were imaged by laser-scanning microscopy.

**Figure 5:**
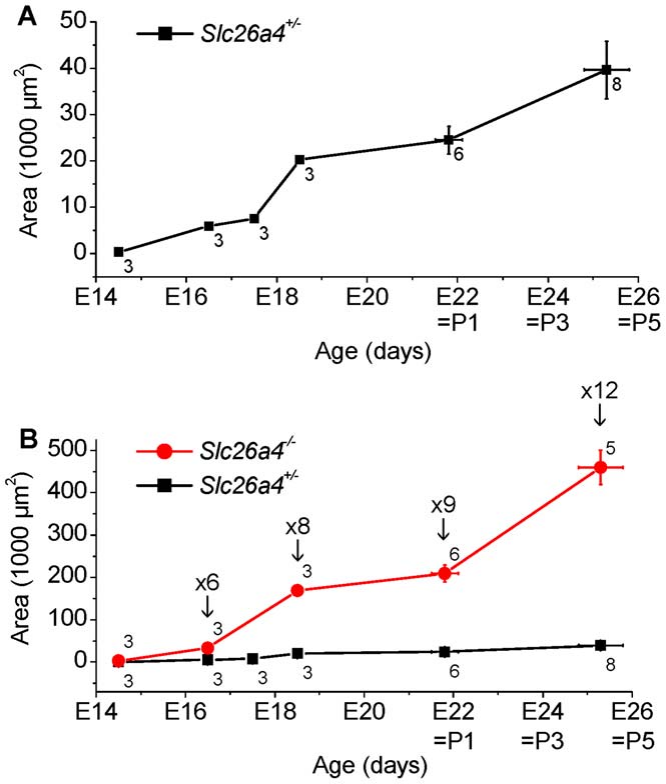
Cochlear lumen development in *Slc26a4^+/−^* and *Slc26a4^−/−^* mice. A, lumen development in *Slc26a4^+/−^* mice. B, lumen development in *Slc26a4^−/−^* mice. Numbers next to symbols represent the N number of *Slc26a4^+/−^* and *Slc26a4^−/−^* littermates. Figure preceded by ‘x’ indicate the factor between measurements in *Slc26a4^+/−^* and *Slc26a4^−/−^* littermates.

In a second set of experiments, we compared the onset of cochlear lumen formation in *Slc26a4^+/−^* and *Slc26a4^−/−^* mice and monitored the progression of the enlargement between E14.5 and ∼P4.5. Lumen formation at E14.5 was advanced in *Slc26a4^−/−^* mice compared to *Slc26a4^+/−^* littermates (Fig. 4). The cochlear lumen increased with development in *Slc26a4^+/−^* and *Slc26a4^−/−^* mice (Fig. 5). The ratio between the luminal size in *Slc26a4^+/−^* and *Slc26a4^−/−^* increase from factor 6 at E16.5 to factor 12 at E18.5.

### Fluid secretion in the vestibular labyrinth ‘pumps up’ the cochlea during lumen formation

**Figure 6:**
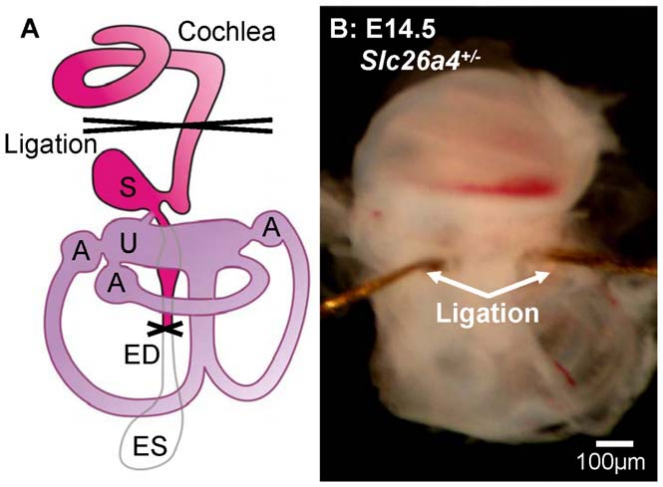
Isolation of the cochlea from the vestibular labyrinth by ligation. A: Diagram of the inner ear. Abbreviations: S, saccule; U, utricle; A, Ampulla; ED, endolymphatic duct; ES, endolymphatic sac. B: Photograph of a ligated inner ear obtained from an E14.5 *Slc26a4^+/−^* mouse.

**Figure 7:**
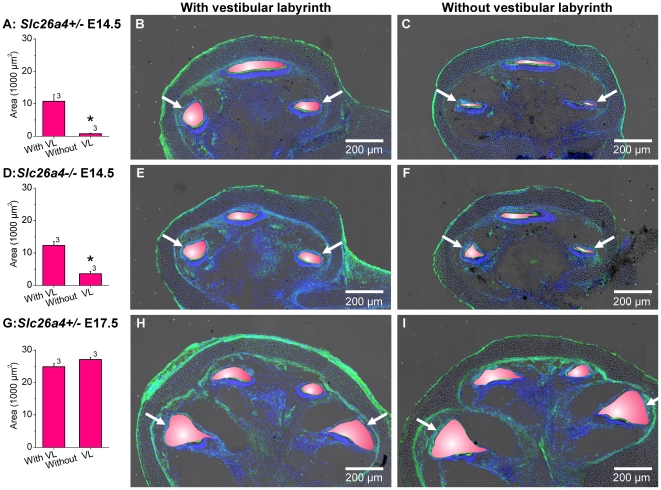
Fluid secretion in the vestibular labyrinth ‘pumps up’ the cochlea during lumen formation. A, D and G: Luminal area measurements (average of the two basal cross-sections) in cochleae with and without vestibular labyrinth. Cochleae were harvested at E14.5 and E17.5 from *Slc26a4^+/−^* and *Slc26a4^−/−^* mice and maintained two days in organ culture. The number next to the bars represents the N number of animals. Significant differences are marked (*). B and C, cochlear cross-sections of E14.5 *Slc26a4^+/−^* mice with and without vestibular labyrinth. E and F, cochlear cross-sections of E14.5 *Slc26a4^−/−^* mice with and without vestibular labyrinth. H and I, cochlear cross-sections of E17.5 *Slc26a4^+/−^* mice with and without vestibular labyrinth. Scala media was highlighted in pink. Images without the pink overlay are available in Figure S1 (Supporting information). F-actin (green) and nuclei (blue) were labeled. Cochlear cross-sections were imaged by laser-scanning microscopy. Arrows point to the two basal cross-sections, which were used for digital area measurements.

In a third series of experiments, we determined whether the vestibular labyrinth contributes to cochlear lumen formation. Embryonic inner ears were harvested at E14.5, which is at the onset of lumen formation, and later, at E17.5. The endolymphatic sac was removed and the cochlea was separated from the vestibular labyrinth by ligation (Fig. 6). To ensure complete isolation of the cochlea from the vestibular labyrinth, the latter was removed micro-surgically after ligation. Ligated and non-ligated inner ears were maintained in organ culture for two days, then sectioned and examined by confocal microscopy. Scala media of inner ears harvested at E14.5 from *Slc26a4^+/−^* mice was found to be significantly smaller in ligated compared to non-ligated preparations (Fig. 7). Similar observations were made in inner ears harvested at E14.5 from *Slc26a4^−/−^* mice. In contrast, no difference in the size of scala media was found between ligated and non-ligated inner ears harvested at E17.5. These observations suggest that the onset of cochlear lumen formation depends in *Slc26a4^+/−^* and *Slc26a4^−/−^* mice on fluid secretion by the vestibular labyrinth. This dependency is transient and was found to be lost at E17.5.

Ligation of the vestibular labyrinth can be expected to isolate the cochlea from the vestibular labyrinth and the endolymphatic sac. The endolymphatic sac was removed in this series of experiments to avoid variations in the preparations due to injury of this extremely fragile structure. The question, whether the endolymphatic sac affects the cochlear lumen was addressed in the next series of experiments.

### Fluid absorption in the endolymphatic sac ‘drains’ the cochlea during lumen formation

**Figure 8:**
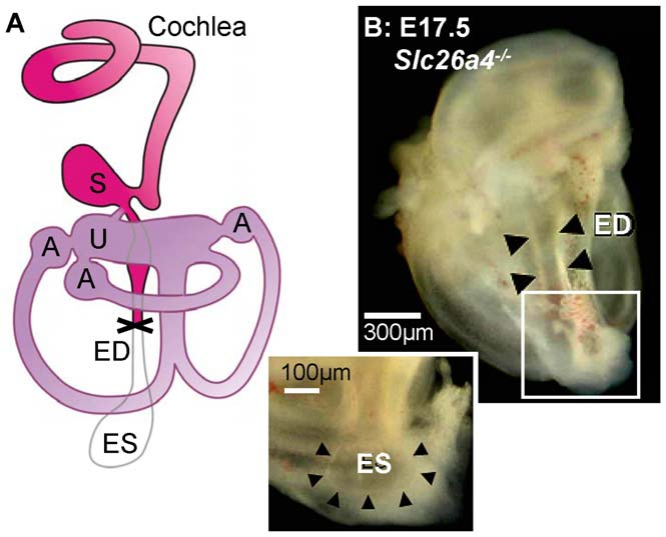
Resection of the endolymphatic sac. A: Diagram of the inner ear. B: Photograph of an inner ear obtained from an E17.5 *Slc26a4^−/−^* mouse. Abbreviations: S, saccule; U, utricle; A, Ampulla; ED, endolymphatic duct; ES, endolymphatic sac.

**Figure 9:**
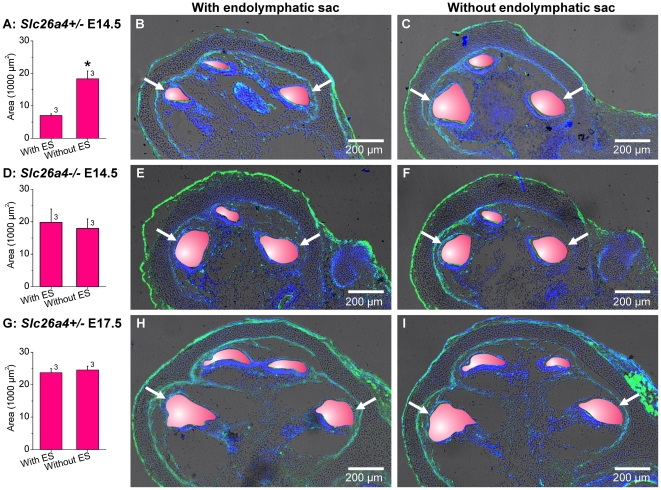
Fluid absorption in the endolymphatic sac ‘drains’ the cochlea during lumen formation. A, D and G: Luminal area measurements (average of the two basal cross-sections) in cochleae with and without endolymphatic sac. Cochleae were harvested at E14.5 and E17.5 from *Slc26a4^+/−^* and *Slc26a4^−/−^* mice and maintained two days in organ culture. The number next to the bars represents the N number of animals. Significant differences are marked (*). B and C, cochlear cross-sections of E14.5 *Slc26a4^+/−^* mice with and without endolymphatic sac. E and F, cochlear cross-sections of E14.5 *Slc26a4^−/−^* mice with and without endolymphatic sac. H and I, cochlear cross-sections of E17.5 *Slc26a4^+/−^* mice with and without endolymphatic sac. Scala media was highlighted in pink. Images without the pink overlay are available in Figure S1 (Supporting information). F-actin (green) and nuclei (blue) were labeled. Cochlear cross-sections were imaged by laser-scanning microscopy. Arrows point to the two basal cross-sections, which were used for digital area measurements.

In a fourth series of experiments, we determined whether the endolymphatic sac contributes to cochlear lumen formation. Embryonic inner ears were again harvested at E14.5 and E17.5. Great care was taken to not injure the endolymphatic sac during dissection. The endolymphatic sac was then removed by manual resection (Fig. 8). Resected and non-resected inner ears were maintained in organ culture for two day, then sectioned and examined by confocal microscopy. The cochlear lumen of inner ears harvested at E14.5 from *Slc26a4^+/−^* mice was found to be significantly larger in resected compared to non-resected inner ears (Fig. 9). In contrast, the cochlear lumen of inner ears harvested at E14.5 from *Slc26a4^−/−^* mice was enlarged and no difference was apparent between resected and non-resected inner ears. Further, no difference in the cochlear lumina were found between resected and non-resected inner ears harvested at E17.5. These observations suggest that the onset of cochlear lumen formation is controlled in *Slc26a4^+/−^* mice by fluid absorption in the endolymphatic sac. This control is transient and found to be lost at E17.5. The observed enlargement in *Slc26a4^−/−^* suggests that loss of *Slc26a4* expression impairs fluid absorption in the endolymphatic sac.

## Discussion

The most salient findings of this study are 1) that lumen formation begins between E13.5 and E14.5 at the base of the cochlea, 2) that the cochlea lumen is transiently, prior to E17.5, controlled by fluid secretion in the vestibular labyrinth and absorption in the endolymphatic sac and 3) that loss of pendrin expression leads to an enlargement of the cochlear lumen that is similar to the enlargement caused by resection of the endolymphatic sac.

The inner ear develops from an invagination of the ectoderm that forms a vesicular otocyst [22]. At ∼E10.5, two protrusions begin to extend from the otocyst; one forms the cochlea and the other forms the endolymphatic sac. While the protrusions elongate and coil, in case of the cochlea, the center of the otocyst reorganizes into the vestibular labyrinth. Pendrin is an anion exchanger that is prominently expressed in the cochlea, the vestibular labyrinth and the endolymphatic sac. In the postnatal cochlea, pendrin is expressed in a spiraling sheet of outer sulcus and spindle cells located in the lateral wall [14]. In the vestibular labyrinth, pendrin is expressed in sheets of transitional cells that surround sensory cell patches, and in the endolymphatic sac, pendrin is expressed in mitochondrial-rich cells that are interspersed among the principal ribosomal-rich cells [11], [14], [23]. The onset of pendrin expression differs among the different compartments of the inner ear. In the endolymphatic sac the onset is at E13 and in the vestibular labyrinth and the cochlea at E15 [24]. A dysfunction of fluid transport due to a lack of pendrin expression can be expected to occur after but is not likely to occur before the onset of expression. The enlargement of the cochlear lumen at E14.5 is therefore unlikely due to local cochlear fluid transport. The relationship been the onset of expression and the onset of cochlear enlargement suggests an involvement of the endolymphatic sac. A reduction in fluid absorption in the endolymphatic sac presents a conceivable reason for the enlargement of the cochlear lumen. This view is supported by the finding that resection of the endolymphatic sac in *Slc26a4^+/−^* mice led to an enlargement similar to that observed in *Slc26a4^−/−^* mice.

**Figure 10:**
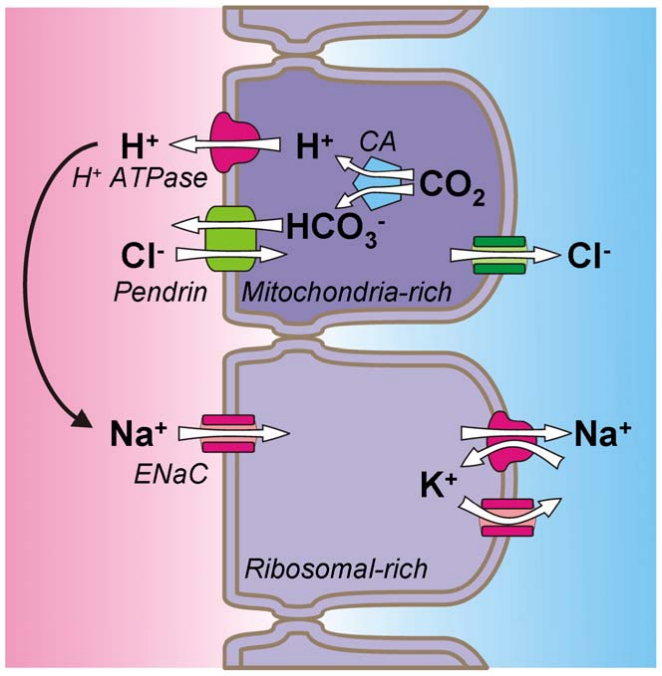
Diagram of ion transport in the endolymphatic sac. The endolymphatic sac epithelium consists mainly of ribosomal-rich cells that are interspersed by mitochondrial-rich cells. Mitochondrial-rich cells express H^+^ ATPase and the Cl^−^/HCO_3_^−^ exchanger pendrin in their apical membrane. Ribosomal-rich cells express Na^+^ channels including ENaC. A current generated by the H^+^ ATPase drives Na^+^ reabsorption via Na^+^ channels. The role of the Cl^−^/HCO_3_^−^ exchanger pendrin is to export HCO_3_^−^ that is generated by carbonic anhydrase (CA) in the reaction that leads to the generation of H^+^.

The concept that the endolymphatic sac is engaged in fluid absorption is not new although a direct, *in vitro*, demonstration of fluid absorption is so far lacking. Indirect evidence for an absorptive function comes from the observation that anoxia leads to a rise in the luminal Na^+^ and Cl^−^ concentrations [25], [26] and that obliteration of the endolymphatic sac in adult guinea pigs to an enlargement of the cochlear lumen [27]. Investigations of the endolymphatic sac are hampered by the difficulty of access and the heterogeneity of the epithelium, which consists principally of ribosome-rich cells with interspersed mitochondrial-rich cells [28]. Nevertheless, a number of ion transporters have been identified. These include apical Na^+^ channels [29], [30], apical H^+^ ATPase subunits [23], [31], [32], basolateral Na^+^/K^+^ ATPases [33], basolateral Na^+^/H^+^ exchangers [34], [35] and apical as well as basolateral Cl^−^/HCO_3_^−^ exchangers including pendrin [11], [23], [31]. The epithelium generates a lumen-positive transepithelial potential that is sensitive to inhibition of Na^+^ channels, H^+^ ATPase, Cl^−^/HCO_3_^−^ exchangers, Na^+^/H^+^ exchangers and carbonic anhydrase [25], [36]. It is intriguing to speculate that the heterogenic epithelium of the endolymphatic sac resembles a frog skin in that it engages in H^+^ ATPase-driven Na^+^ reabsorption. Frog skin is comprised of principal cells equipped with apical Na^+^ channels and mitochondria-rich cells equipped with an apical H^+^ ATPase and a Cl^−^/HCO_3_^−^ exchanger. Highly effective Na^+^ absorption is powered by the H^+^ ATPase, which generates a current that drives Na^+^ absorption through the Na^+^ channels [37]. The role of the Cl^−^/HCO_3_^−^ exchangers is this model is to export HCO_3_^−^ that is generated by carbonic anhydrase in the process of generating H^+^ as substrate for the H^+^ ATPase (Fig. 10). Lack of the Cl^−^/HCO_3_^−^ exchanger pendrin would inhibit H^+^ production by carbonic anhydrase through product-inhibition and a reduce the current that drives Na^+^ reabsorption.

The observation that resection of the endolymphatic sac caused an enlargement at E14.5, but not at E17.5, is intriguing. The loss of control of cochlea fluid homeostasis by the endolymphatic sac may be the result of the maturation of local cochlear fluid homeostasis. Further, the capacity for Na^+^ reabsorption in the endolymphatic sac may be higher at E14.5 than at E17. Support for this hypothesis comes from the finding that numbers of mRNA copies coding for the three subunits of the Na^+^ channel ENaC decline from P2 to P6 of early postnatal development, which is ∼4 days after E17 [29]. It is conceivable that this decline can be extrapolated to the embryonic phase of development.

Lumen formation was observed in the cochlea to progress from base to apex. A base-to-apex gradient in developmental maturation is consistent with the cochlea developing from an elongating protrusion of the otocyst, which implies that cells near the base are developmentally more mature than cells at the apex. Cochlear lumen formation could thus be due to a base-to-apex progressing onset of local fluid transport. Alternatively, lumen formation could be aided by the vestibular labyrinth, which matures earlier than the cochlea. For example, the onset of mechanotransduction in vestibular hair cells is at E17, which is ∼4 days earlier than in the cochlea [38], [39]. The observation that ligation of the vestibular labyrinth caused a reduction of the cochlear lumen at E14.5 but not at E17.5 suggests that cochlear lumen formation is transiently aided by fluid secretion in the vestibular labyrinth. Fluid secretion in the embryonic cochlea or vestibular labyrinth is poorly understood. Mechanism of ion transport, that are well established in the postnatal cochlea, cannot be extrapolated to the embryonic stage since the adult ion composition of the luminal fluid, which is characterized by a high K^+^ concentration and low Na^+^ and Ca^2+^ concentrations, is established only during postnatal development [12], [17], [18]. Nevertheless, the observation that ligation of the vestibular labyrinth led to a reduction in the cochlear lumen in *Slc26a4^+/−^* and *Slc26a4^−/−^* mice suggests that pendrin does not play a major role in fluid secretion in the embryonic vestibular labyrinth.

Studies in *Slc26a4^−/−^* mice have provided a basis to understand the spectrum of phenotypes seen in human patients that range from deafness at birth to deafness during childhood. Mice lacking pendrin have been shown to develop a ∼10-fold enlargement of the cochlear lumen. This enlargement forms during embryonic development and persists throughout postnatal life [9], [10], [14].

During the developmental phase, the enlargement may disrupt cell-to-cell signaling that is necessary for the development of hearing. Gap junction mediated signaling between epithelial cells, for example, may be compromised by increased diffusional distances that are inherent to stretched epithelial cells enclosing an enlarged lumen (Fig. 1B). The importance of gap junction mediated cell signaling is underlined by the fact that loss-of-function mutations of the gap junction protein connexin 26 cause deafness at birth. Coincidentally, mutations of connexin 26 are the most frequent cause for non-syndromic hearing loss in children [40], [41]. Further, the enlargement may disrupt signaling between mesenchymal and epithelial cells as shown in the early postnatal cochlea where mesenchymal cells generate thyroid hormone by conversion of the prohormone to the hormone and epithelial cells bear the thyroid hormone receptors that control proper maturation of the cochlea [42], [43], [44], [45]. The enlargement of the cochlea lumen in *Slc26a4^−/−^* mice displaces mesenchymal cells and thereby disrupts cell signaling, which leads to local cochlear hypothyroidism during the early postnatal phase of development [10]. Taken together, cell-to-cell signaling is critically important during development of the cochlea. The enlargement appears to disrupt critical cell-to-cell signaling mechanisms at multiple stages, thereby disrupt proper development and account for deafness at birth in mice and possibly human patients.

Many human patients bearing loss-of-function mutations of *SLC26A4* are born hearing, which suggests that cochlear development in humans may to some extent escape the detriment of hampered cell-to-cell signaling during early development. The majority of patients bearing mutations of *SLC26A4* lose hearing during early childhood - often after a period of fluctuating hearing loss that progresses toward deafness [8], [46]. Based on studies in the *Slc26a4^−/−^* mouse model, fluctuating hearing loss may be due to oscillations of a negative feedback system that oscillates and generates fluctuations in the endocochlear potential and thereby in the driving force on which hearing depends. This feedback loop is comprised of reactive oxygen species (ROS) generated by marginal cells of stria vascularis as a byproduct of elevated rates of K^+^ secretion and the ROS-sensitive K^+^ channel *KCNJ10* that generates the endocochlear potential and supplies K^+^ to the marginal cells [12], [13]. The ROS-induced loss of *KCNJ10* abolishes the endocochlear potential and hearing and the associated reduction in K^+^ flux to marginal cells would limit K^+^ secretion, reduce ROS production, allow restoration of *KCNJ10* expression, restoration of the endocochlear potential and restoration of hearing. This feedback loop may oscillate and thereby generate fluctuating hearing loss. Irreversible deafness would ensue, when endolymphatic Ca^2+^ concentrations rise and hair cells succumb to Ca^2+^ overload [9], [12].

In summary, the present study demonstrates that a lack of functional *Slc26a4* expression leads to a failure of fluid absorption in the endolymphatic sac that initiate cochlear enlargement in mice, and possibly humans. It is conceivable that deafness in mice, and possible humans, is a direct consequence of the cochlear enlargement and inherent disruption of cell signaling and cochlear ion homeostasis.

## Supporting Information

Figure S1 Cochlear lumen formation in *Slc26a4+/−* and *Slc26a4−/−* mice. These images correspond to Fig. 4 in the main manuscript.
(7.04 MB TIF)

Figure S2 Fluid secretion in the vestibular labyrinth ‘pumps up’ the cochlea during lumen formation. These images correspond to Fig. 7 in the main manuscript.
(7.02 MB TIF)

Figure S3 Fluid absorption in the endolymphatic sac ‘drains’ the cochlea during lumen formation. These images correspond to Fig. 9 in the main manuscript.
(8.66 MB TIF)
