# Supplementary figures and images for: Failure of Fluid Absorption in the Endolymphatic Sac Initiates Cochlear Enlargement that Leads to Deafness in Mice Lacking Pendrin Expression
Source: PLoS One. 2010 Nov 17;5(11):e14041. doi: 10.1371/journal.pone.0014041 (PMC2984494; doi:10.1371/journal.pone.0014041)

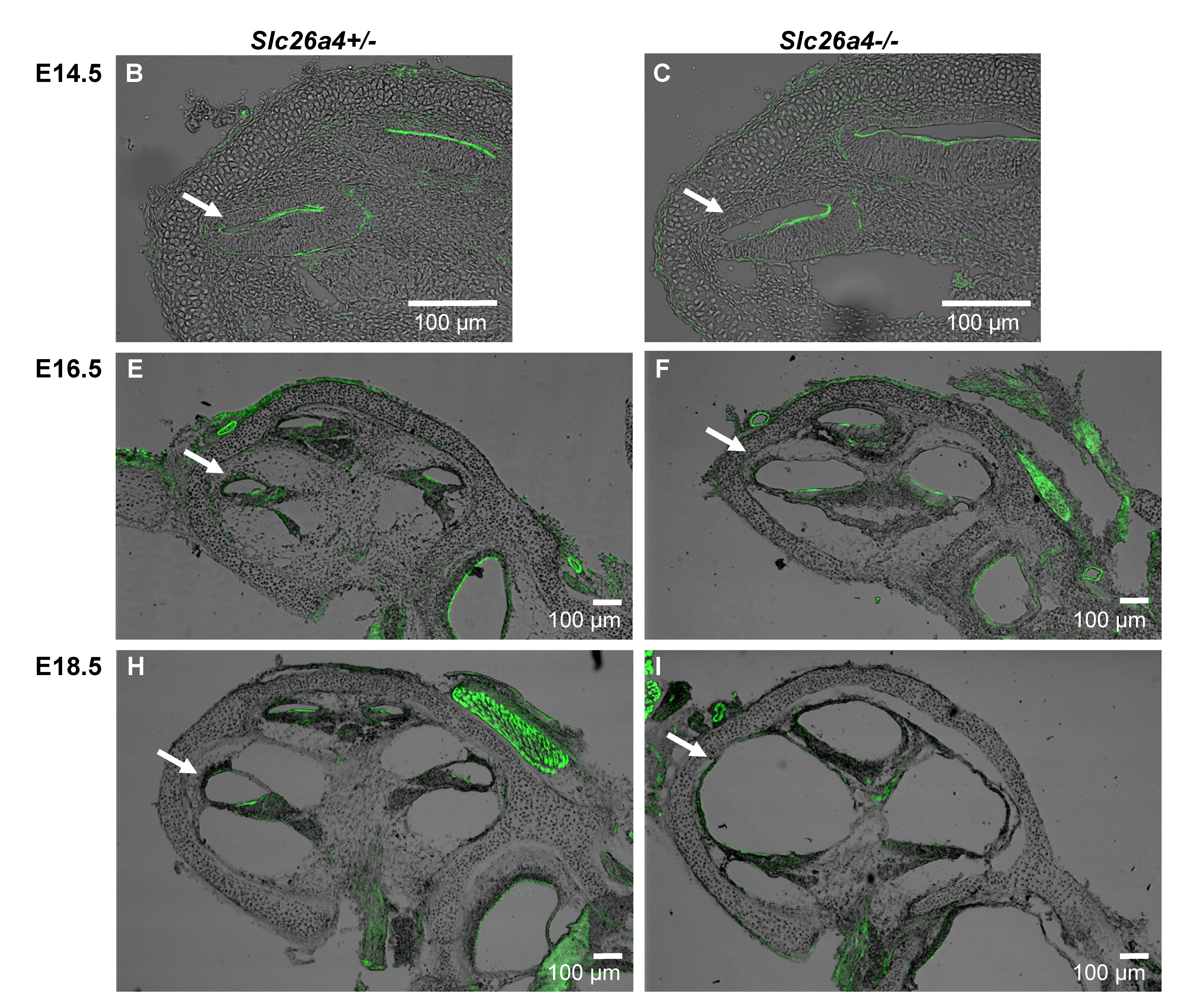

Supplement: Figure S1 — Cochlear lumen formation in Slc26a4+/− and Slc26a4−/− mice. These images correspond to Fig. 4 in the main manuscript. (7.04 MB TIF) [file pone.0014041.s001.tif]

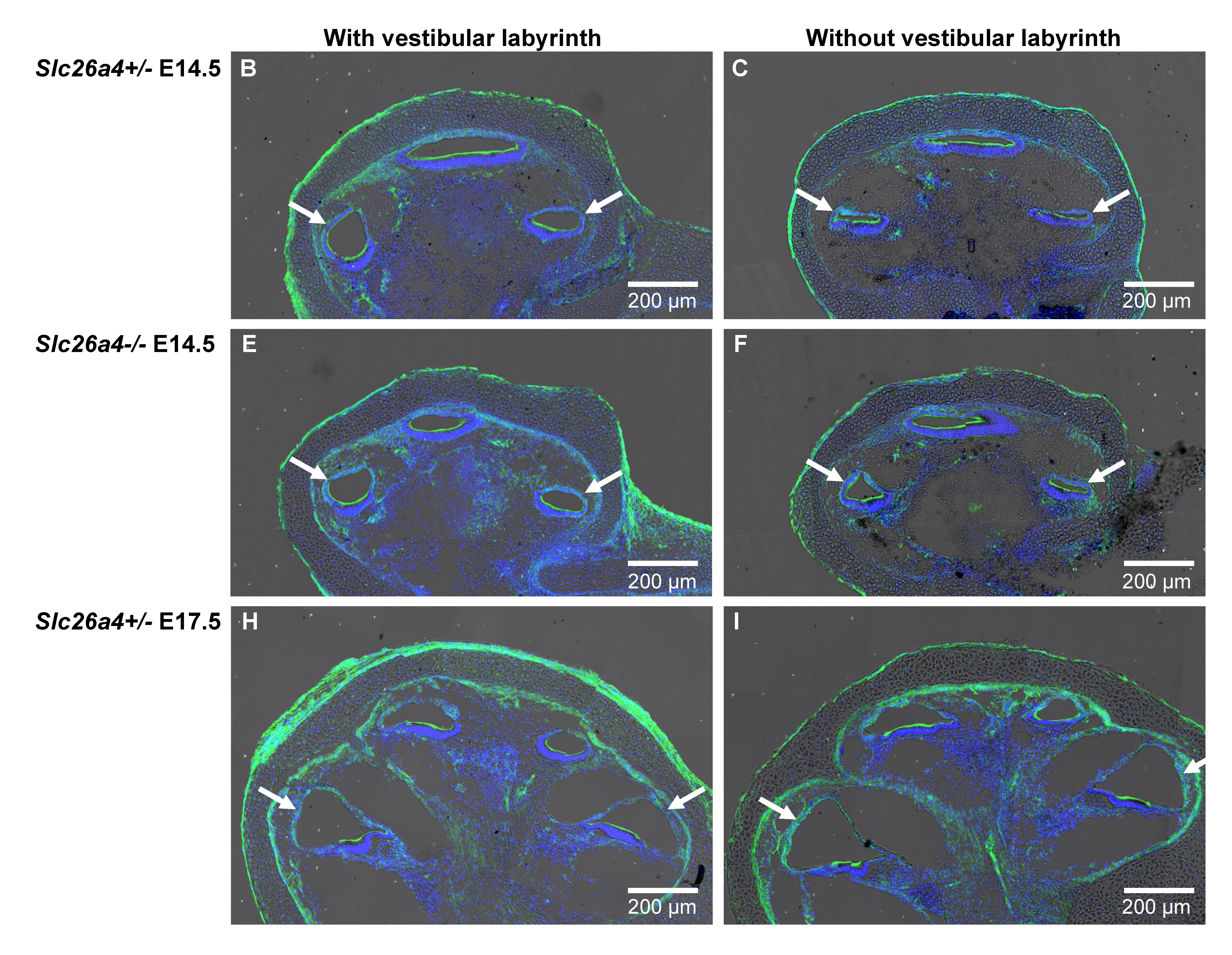

Supplement: Figure S2 — Fluid secretion in the vestibular labyrinth ‘pumps up’ the cochlea during lumen formation. These images correspond to Fig. 7 in the main manuscript. (7.02 MB TIF) [file pone.0014041.s002.tif]

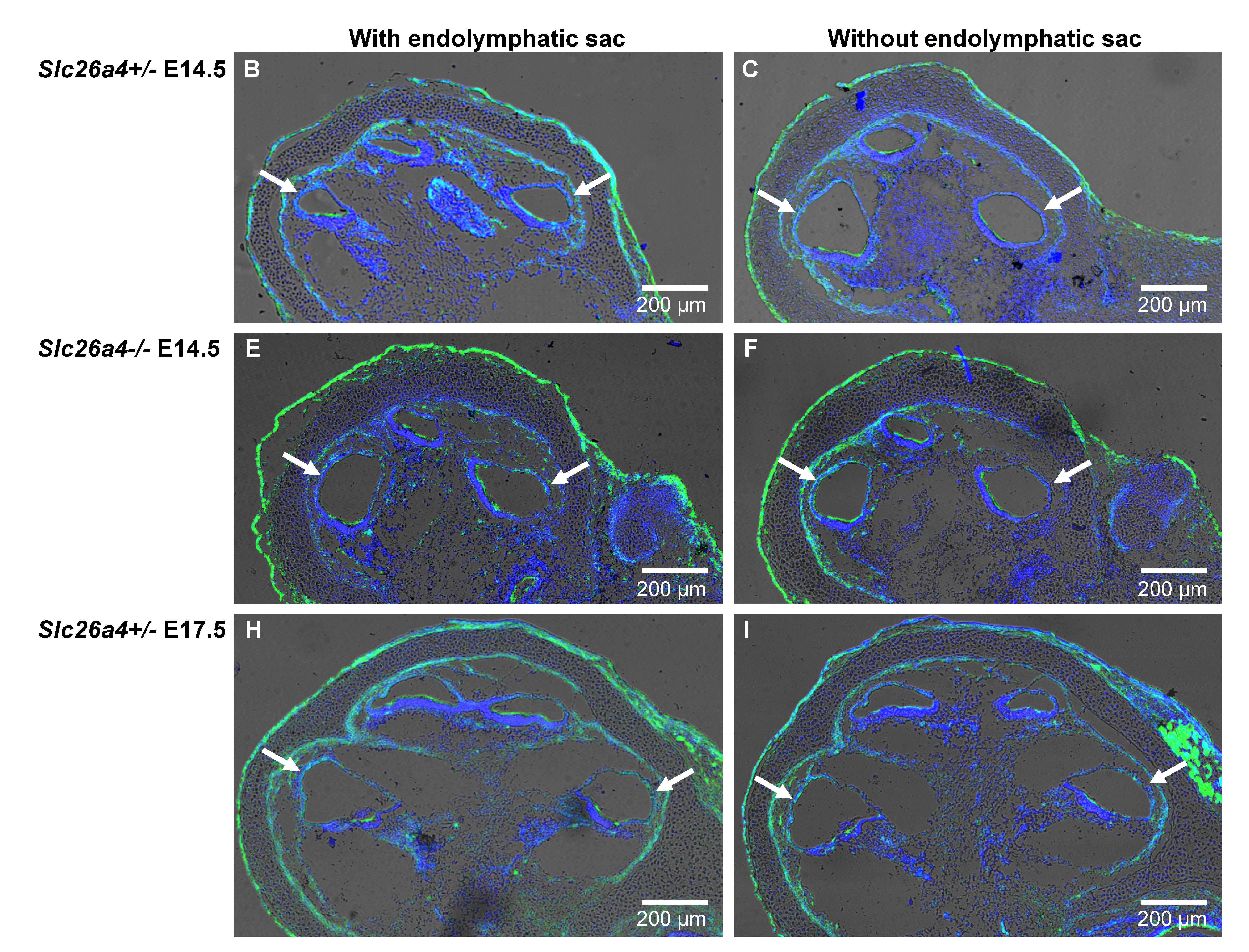

Supplement: Figure S3 — Fluid absorption in the endolymphatic sac ‘drains’ the cochlea during lumen formation. These images correspond to Fig. 9 in the main manuscript. (8.66 MB TIF) [file pone.0014041.s003.tif]
